# The role of polio emergency operations centers: perspectives for future disease control initiatives in Nigeria

**DOI:** 10.11604/pamj.supp.2023.45.2.41308

**Published:** 2023-08-08

**Authors:** Fiona Braka, Usman Adamu, Anis Siddique, Omotayo Bolu, Eunice Damisa, Richard Banda, Sume Gerald, Charles Korir, Samuel Usman, Aminu Mohammed, Shina Aladeshawe, Sisay Tegegne, Terna Nomhwange, Endie Waziri, Patrick Nguku, Tesfaye Erbeto, Peter Nsubuga, Faisal Shuaib

**Affiliations:** 1 World Health Organization Country Office, Abuja, Nigeria,; 2 National Primary Health Care Development Agency, Abuja, Nigeria,; 3 United Nations Children Education Fund, Abuja, Nigeria,; 4 United States Centers for Disease Control and Prevention, Abuja, Nigeria,; 5 Core Group of Partners, Abuja, Nigeria,; 6 Rotary International, Lagos, Nigeria,; 7 Bill and Melinda Gates Foundation, Abuja, Nigeria,; 8 Africa Field Epidemiology Network, Abuja, Nigeria,; 9 Global Public Health Solutions, Atlanta Georgia, United States

**Keywords:** Polio eradication, emergency operations center, wild poliovirus, transition

## Abstract

The Nigeria Polio Emergency Operations Centre (EOC) was established in October 2012 to strengthen coordination, provide strategic direction based on real-time data analysis, and manage all operational aspects of the polio eradication program. The establishment of seven state-level polio EOCs followed. With success achieved in the interruption of wild poliovirus (WPV) transmission as certified in 2020, the future direction of the polio EOC is under consideration. This paper describes the role of the polio EOC in other emergencies and perspectives on future disease control initiatives. A description of the functionality and operations of the polio EOC and a review of documentation of non-polio activities supported by the EOC was done. Key informant insights of national and state-level stakeholders were collected through an electronic questionnaire to determine their perspectives on the polio EOC’s contributions and its future role in other public health interventions. The polio EOC structure is based on an incident management system with clear terms of reference and accountability and with full partner coordination. A decline in WPV1 cases was observed from 122 cases in 2012 to 0 in 2015; previously undetected transmission of WPV1 was confirmed in 2016 and all transmission was interrupted under the coordination of the EOCs at national and state levels. During 2014-2019, the polio EOC infrastructure and staff expertise were used to investigate and respond to outbreaks of Ebola, measles, yellow fever, and meningitis and to oversee maternal and neonatal tetanus elimination campaigns. The EOC structure at the national and state levels has contributed to the positive achievements in the polio eradication program in Nigeria and further in the coordination of other disease control and emergency response activities. The transition of the polio EOCs and their capacities to support other non-polio programs will contribute to harnessing the country’s capacity for effective coordination of public health initiatives and disease outbreaks.

## Introduction

The 65th session of the World Health Assembly declared Polio Eradication a programmatic emergency for global public health in May 2012 [1]. By 2012, Nigeria was Africa’s only polio-endemic country. Despite efforts to curtail polio outbreaks, the number of new wild poliovirus type 1 (WPV1) cases in Nigeria increased sharply from 21 in 2010 to 62 in 2011 to 122 in 2012, and WPV type 3 transmission persisted [2]. The increasing trend in WPV cases was attributable to weak program management, poor quality campaign performance, poor coordination of activities among government and partners, lack of ownership and accountability at all levels of the program, and lack of community support for polio vaccination activities; there were parental concerns about vaccine safety resulting in a high level of non-compliance and mistrust at the community level [3]. A National Polio Eradication Initiative (PEI) coordination mechanism had been in existence at the National Primary Health Care Development Agency (NPHCDA) for approximately 10 years within the Department of Disease Control and Immunization. However, the structure was mainly functional during supplemental immunization activities (SIAs) and staffed primarily by NPHCDA staff, with occasional support provided by partner agencies.

The Presidential Task Force on Polio Eradication (PTFoPE), inaugurated in March 2012, commissioned a National Polio Emergency Operations Center (EOC) in October 2012 in Abuja. The EOC serves as the operational arm and Secretariat of the PTFoPE and is responsible for providing strategic direction and managing all operational aspects of the program [3]. The EOC model required government and Global Polio Eradication Initiative (GPEI) partners to deploy their senior and experienced staff to one location, under the government’s leadership, to plan, execute and monitor eradication strategies and deliberate strategies and tactics jointly. The EOC is expected to promote a common framework for joint problem identification and solving; timely analysis, interpretation, and dissemination of data and information; enforce accountability for results; and enhance a coordinated response to evolving situations. Seven State EOCs were additionally established in 2012 along a similar structure to coordinate state-level efforts in select high-risk states (Bauchi, Borno, Kaduna, Kano, Katsina, Sokoto and Yobe) in northern Nigeria. Support was provided by the Bill and Melinda Gates Foundation (BMGF) to set-up and maintain facilities accommodating the national and state EOCs.

The EOCs have been credited with driving the remarkable successes observed in the Nigeria polio eradication program [4]. The number of WPV1 cases dropped to zero between 2012 and 2015. When previously undetected, persistent WPV1 transmission was identified in Borno State in northeast Nigeria among children from areas outside the reach of the polio program for several years due to the Boko Haram insurgency. The EOC swiftly implemented a robust coordinated response to this finding and devised innovative solutions to vaccinate children in a majority of insecure areas and search for potential continued transmission in those areas. The EOC has provided a platform for coordination and support to other public health initiatives, including using the polio EOC structure and personnel for the Ebola outbreak response in 2014 [5,6].

By September 2019, the country marked 3 years with no isolation of WPV1 with enhanced immunization and surveillance activities in insecure areas, paving the way for the declaration of interruption of transmission in Nigeria and certification of the World Health Organization (WHO) Africa Region as free of indigenous WPV transmission by the African Regional Certification Commission for Polio Eradication (ARCC) on 25 August 2020. With success achieved, and although essential polio eradication functions must continue, the polio EOCs are under consideration for transitioning to other emergency functions, along with the range of polio human resources and logistical assets built over the years. We report an assessment that aims to document the role of the polio EOCs in the coordination of broader public health programs and perspectives for future roles in disease control initiatives; this can inform decision-makers on the options for the transition of polio assets in Nigeria.

## Methods

**Study design:** we examined the organization and structure of the polio EOCs at the Federal and State level and the respective responsibilities in 2019. We obtained information on the annual operational costs for the EOCs from a 2019 National Polio Asset Mapping Report, from a review by NPHCDA and partners. We reviewed polio reports summarized from the national polio database. Likewise, we assessed records and reports on non-polio-related activities coordinated by the EOCs. Key informant interviews were conducted to obtain information on the role of the polio EOCs in the coordination of non-polio-associated activities during 2014-2019 and future perspectives after certification of WPV1-free status.

**Setting:** the assessment was implemented at the national level (Polio EOC, Nigeria Centre for Disease Control [NCDC], GPEI partners) and state level (Polio EOCs and State Primary Health Care Development Agencies [SPHCDAs]).

**Organization of the emergency operations centre**

**Figure 1:**
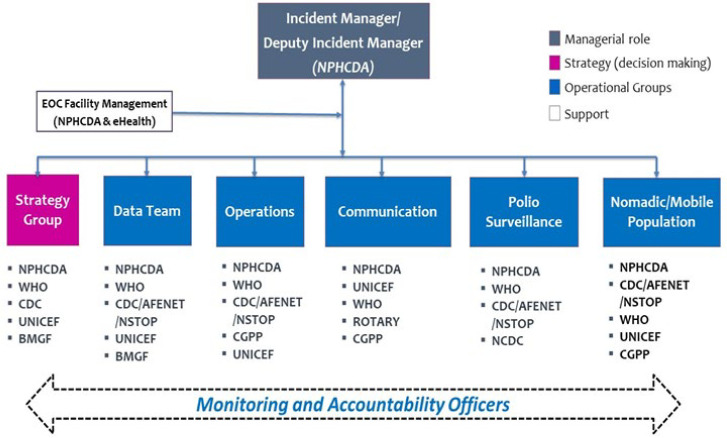
organogram of the National Polio Emergency Operations Centre (EOC), Nigeria

We examined the organization and structure under the incident management system (IMS) which includes an Incident Manager and Deputy Incident Manager designated by NPHCDA with the authority to execute decisions related to the program on behalf of the Executive Director of NPHCDA. Similarly, State EOCs set up in seven states (i.e., Borno, Kaduna, Kano, Katsina, Sokoto, Bauchi, and Yobe) have designated Incident Managers and Deputy Incident Managers from the SPHCDA. The National EOC structure has five subgroups that facilitate the functions of the EOC: Strategy, Data Management, Operations, Communication, and Polio Surveillance (with a small subgroup also focusing on mobile populations), and, supported by Monitoring and Accountability (M and A) Officers and a facility management team (Figure 1). The emergency operations centre has five operational components:

***1) War-room approach:*** a layout facilitating the display of maps, up-to-date polio program performance indicators, and video and teleconference facilities that link with the state EOCs. The EOC members meet twice a week in the war room, while the subgroups of the EOC hold separate meetings during the week. However, during outbreaks, the number of meetings may increase in frequency.

***2) Dedicated cross-functional talent:*** the National Primary Health Care Development Agency provides the leadership team for the National EOC and more than 50 M and A Officers with direct contact to states and high-risk LGAs. WHO and the Nigeria Office of the US Centers for Disease Control and Prevention (CDC) provide polio experts, epidemiologists and data analysts, while UNICEF provides expertise to address communication, social mobilization, non-compliance, and logistics management. WHO, UNICEF, CDC (through its implementing partner Africa Field Epidemiology Network(AFENET)/ National Stop Polio (NSTOP)Program and Core Group of Polio Partners (CGPP) deploy thousands of ‘boots on the ground’ to support state and local governments right down to community level in implementing and monitoring polio eradication activities, including surveillance and mobilization.

***3) Fast-paced analytics and frequent synthesis:*** the EOC analyses data periodically and in real-time for prompt action. Pre-, intra-, and post-vaccination campaign data are analyzed and displayed in monitoring dashboards, and strategies to improve SIA quality are systematically evaluated.

***4) Rapid decision-making and synthesis:*** the EOCs ensure that all relevant decision-makers are under one roof to review performance data from the field and make decisions rapidly. There is delegated authority from the NPHCDA to the Incident Manager to execute decisions of the EOC.

***5) Intensive program management:*** rigorous and daily tracking of activities in the field through telephone, satellite imagery, GIS technology, open data kit (ODK), and other mechanisms, as well as overall monitoring of outcomes against clear targets set out in the annual National Polio Eradication Emergency Plan, helps to maintain a sense of urgency at all times.

**Study participants:** key informants from the Federal and State level EOCs were selected from government and partner agencies based on their senior level of responsibility and involvement in coordinating public health programs. Eighteen key informants responded to the interview questionnaire which covered several thematic areas including resources for polio EOC, the role and impact of EOCs on other programs/responses etc (Annex 1). The location (and number) of the participants spanned across the National EOC/ NPHCDA (4), NCDC (2), State EOC (6), State Primary Health Care Development Agency (SPHCDA)/ State Government (2), Partner Agencies (WHO, UNICEF, CDC, BMGF, Rotary International) (4). All participants selected had some degree of involvement with, knowledge of, or exposure to polio eradication activities and participated in the coordination of activities at the Federal or State level. The 18 participants were involved in other program areas: routine immunization (15; 83.3%), communicable disease control (12; 66.7%), emergencies and outbreak response (12; 66.7%), child health (11; 61.1%), maternal health (8; 44.4%), health systems strengthening (7; 38.9%) and operations (7; 38.9%).

**Figure:**
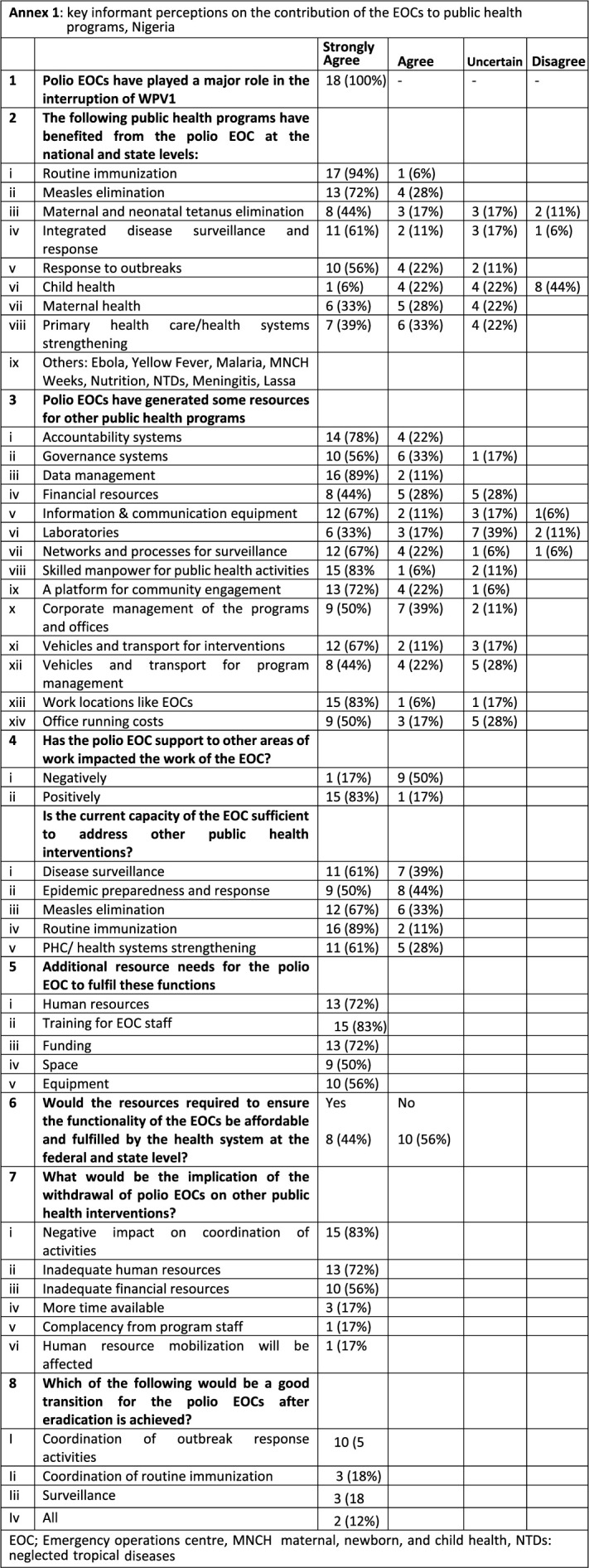


**Data collection and analysis:** we obtained information about the polio EOC, operational costs, and involvement in other disease control efforts from records and published literature. In addition, an electronic questionnaire was administered to key informants at the federal and state level using a survey platform on Google. An open-source data collection tool was designed specifically for the survey, and the responses were anonymous. After downloading the responses in Comma-Separated Values (CSV) format, we generated charts and determined the frequencies of quantifiable responses using Microsoft Excel, and identified common themes for qualitative analysis.

**Ethical considerations:** the assessment was part of the National Polio Transition Plan of the Government of Nigeria, which included documentation of best practices and lessons learned from the polio program to benefit other public health initiatives. We obtained permission from the National EOC to review reports and reference documentation on the contribution of the Polio EOCs. Informed consent was obtained from each survey participant at the start of the questionnaire; consenting participants continued filling out the questionnaire.

## Results

**Figure 2:**
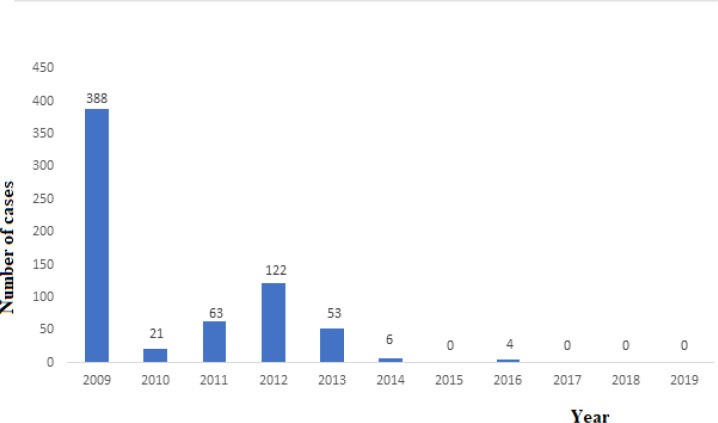
trend in type 1 (WPV1) cases, Nigeria, 2009-2019

**Trend in wild poliovirus incidence:** following the inception of the EOC in 2012, the number of WPV1 cases dropped from 122 in 2012 to 0 in 2015. In August-September 2016, four WPV1 cases were confirmed from Borno State in areas that had not been accessible to the programme for three to four years because of insecurity, with resident populations that were larger than estimated remaining in some areas. Transmission was confirmed in three Local Government Areas (LGAs) of Jere, Gwoza and Monguno in Borno State. An aggressive response was mounted and coordinated by the National EOC and Borno State EOC, which included innovative approaches, such as the use of security personnel to reach children in inaccessible areas (i.e. areas not accessible to vaccination teams) with the oral polio vaccines. The last confirmed case had an onset of paralysis on 21 August 2016 (Figure 2).

**Operational costs of the emergency operations centers:** the national polio EOC is housed on rented premises, while the State governments provide the buildings to house the State EOCs. The management of the EOCs is outsourced by BMGF to a third party (e-Health Africa). Running costs are incurred for utilities, security, cleaning and maintenance services. The total annual cost for the renting and operations of the eight EOCs is estimated at USD 5,625,346.4121.

**Support for non-polio related activities:** the polio EOC structure provides a platform for coordinating the responses to disease outbreaks, particularly related to vaccination response activities for Vaccine preventable diseases (VPDs). Government and partner efforts are coordinated under one roof to facilitate planning, monitoring preparedness for campaigns, tracking intra- and post-campaign activities, managing and analyse data, and providing feedback. During 2014-2019, the country experienced outbreaks of Ebola, meningococcal meningitis, measles, and yellow fever. The polio EOCs served as the location for coordinating the outbreak responses at the federal and state levels. In addition, the polio EOC staff were deployed to support response activities such as surveillance, suspect case investigation, microplanning, implementation, community mobilization, supervision, and monitoring (Annex 2). Furthermore, as part of the country’s efforts to achieve maternal and neonatal tetanus elimination (MNTE), mass vaccination campaigns were conducted in selected high-risk states with the support of the polio infrastructure and EOC coordination.

**Figure:**
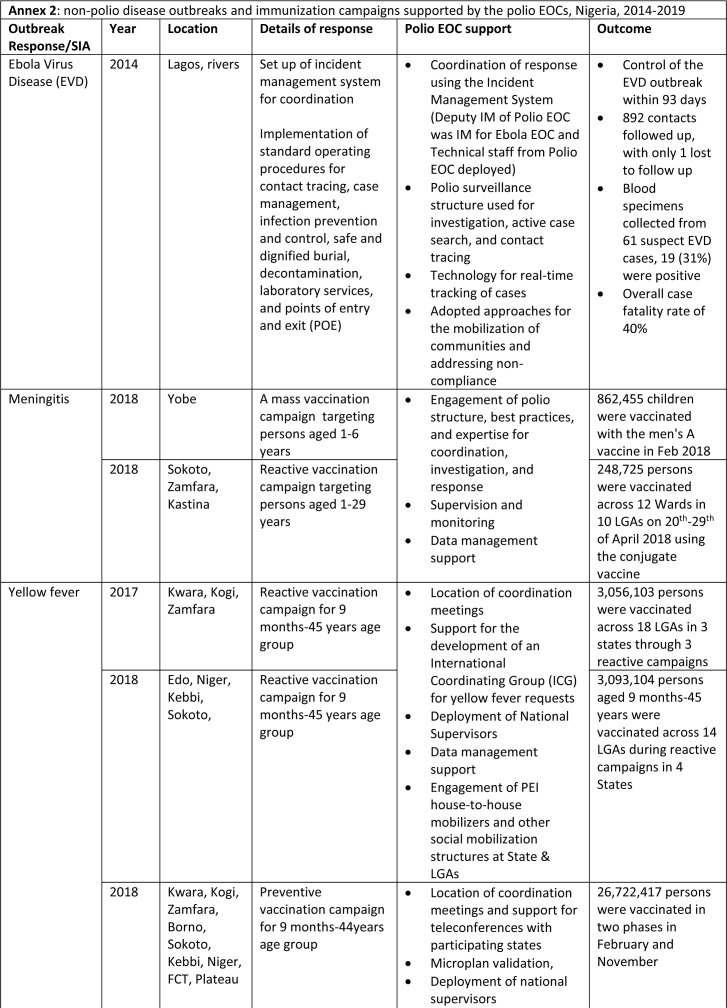

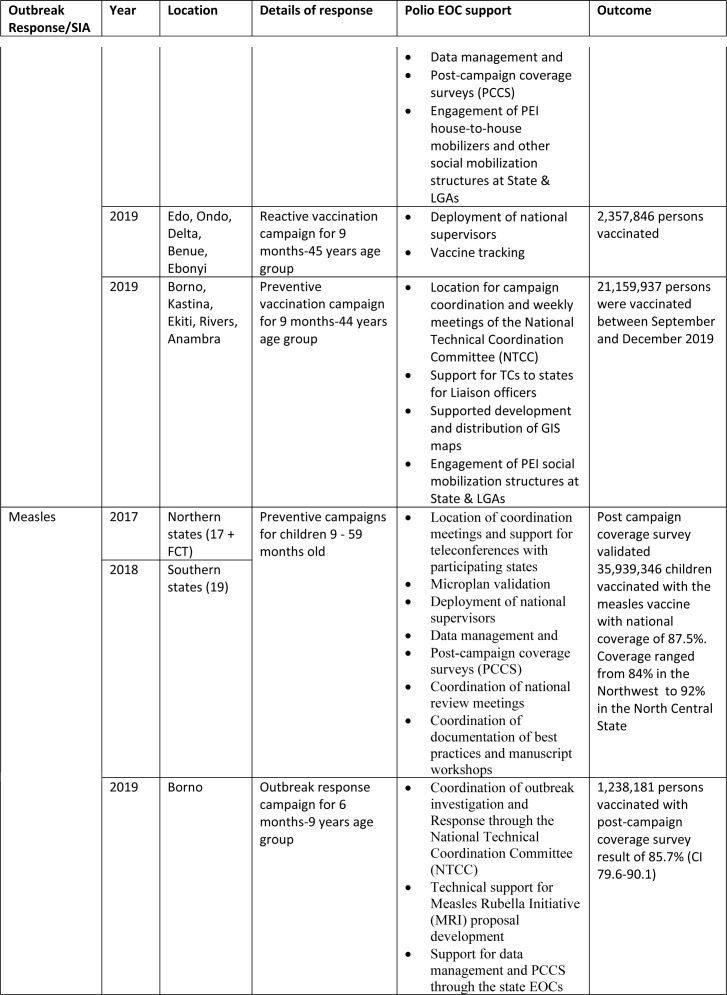

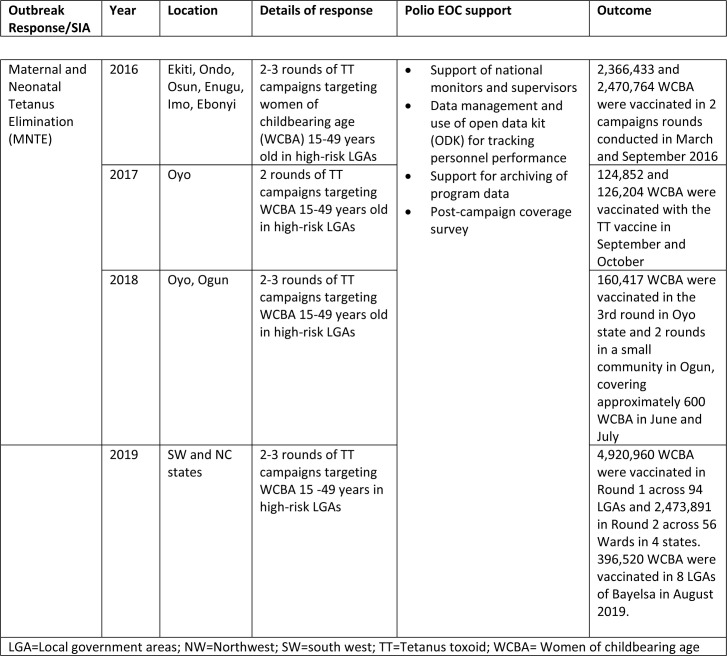


**Informants´ insights:** all key informants (100%) were unanimous in their opinion that the polio EOCs played a significant role in the interruption of WPV1 in Nigeria. Besides polio eradication activities, respondents noted that the Polio EOCs strongly contributed to other public health programs, notably routine immunization strengthening, measles elimination and integrated disease surveillance and response (IDSR). There was some agreement that the EOCs also contributed to MNTE and Primary Health Care (PHC)/ health systems strengthening, while less contribution was perceived towards maternal health and least in child health (Annex 1). The resources at the EOCs were noted by 89% of key informants to have strongly supported data management and analysis; provided workspace for program staff, deployed a skilled workforce for public health systems; implemented accountability systems; provided platforms for proposals to expand community engagement; increased effective use of information and communication technology, vehicles and transport, governance systems, and networks; and strengthened the processes for surveillance. There was some agreement that contributions were also made towards office running costs and corporate management, while the laboratories were perceived to have made a less or uncertain contribution towards other programs.

Over 60% of respondents perceived that the current capacity at the EOCs is enough to address other public health interventions, notably routine immunization strengthening, measles elimination, disease surveillance, and enhancing PHC health systems. In contrast, only half of the respondents agreed the capacity was adequate for epidemic preparedness and response. Additional resources would be needed at the EOCs to effectively meet the needs of other programs, notably in capacity building, human resources, and funding. 56% of respondents felt the resources to meet these needs are not affordable by the government at the federal and state levels and would require external support. Of the 18 respondents, the majority noted that the withdrawal or closure of the EOCs would negatively impact coordination (15; 83%), human resources (13; 72%), and external financial support (10; 56%) for other interventions. Following the achievement of polio eradication, the transition of polio EOCs would be best towards coordination of outbreak response activities (n; 53%), routine immunization (n; 89%), surveillance (n; 61%) or all three areas mentioned (n; 12%).

## Discussion

Key informants involved in Nigeria´s polio efforts unanimously agreed that Polio EOCs played a major role in coordinating and implementing the activities that led to the interruption of WPV transmission and have also provided a platform for coordinating other disease control initiatives in Nigeria, demonstrating the benefit for the transition of the EOCs for future, broader public health use. After the achievement of WPV-free status, the polio EOC is working to interrupt the circulating vaccine-derived poliovirus type 2 (cVDPV2) outbreak, which is currently occurring in Nigeria and several other countries and regions that had achieved WPV free certification. Emergency Operations Centres succeed with clear leadership through an IMS with an Incident Manager and delegated responsible areas, with standardized roles and clear triggers for action. The IMS promotes a unified command structure and decision-making, clear terms of reference, consolidated plans with targets, accountability, integrated communication, and a comprehensive strategy to efficiently manage all resources available to the EOC [8,9]. The polio EOC operation in Nigeria is based on the IMS. The EOCs are government-led, and members (including designated partner representatives) have the decision-making authority to act quickly in implementing a joint plan.

The coordination of the polio program under the EOC structure at federal and state levels and specific intensified efforts at the field level was effective in significantly reducing the burden of WPV in Nigeria. A review of polio eradication initiative (PEI) coordination structures in Angola, DRC, Côte d’Ivoire, and Chad showed that coordination structures in place led to synergistic effects that enabled the polio program to achieve its objectives, with important spill-overs in the coordination of other public health programs [4]. A central coordination mechanism was augmented by subnational structures that decentralized coordination to lower level in all the countries, closest to public health action. The Polio Eradication and Endgame Strategic Plan 2013-2018 placed a strong and clear emphasis on the necessity of improving routine immunization (RI) to achieve and sustain global polio eradication [10]. The plan recognized that as the eradication target approaches, there is a need for GPEI to contribute to strengthening RI towards achieving the last mile in polio eradication by transferring effective elements of the polio program to national immunization programs. The Polio Endgame Strategy 2019-2023 further reinforced the necessity of integration for joint delivery and coordination of PEI with other VPD control and elimination targets, and polio surveillance with VPD surveillance [11]. This was demonstrated in the case of the Polio EOC of Nigeria, with significant contributions made in non-polio VPD outbreak responses. Through systematic collaboration with other public health actors, integrated capacities and contributions beyond GPEI can help achieve and sustain eradication. At the same time, GPEI assets, knowledge, and expertise can be channeled to protect populations by supporting the strengthening of RI, health systems and emergency responses. Other public health programs can benefit from the technical and operational capacity and funding to make substantive program improvements [12]. Many polio eradication assets and lessons learned have already been applied to global and African regional measle’s elimination efforts, as the PEI and measles elimination efforts have similar strategies and program implementation infrastructure needs [13].

In alignment with the GPEI strategy, our assessment demonstrated that the Nigeria polio EOCs´ capacities were successfully re-purposed to support other health emergencies such as outbreaks of Ebola, measles, yellow fever, and meningitis, and towards strengthening the immunization system. Our findings align with previous reports that documented the positive contribution of the polio EOCs in other disease control initiatives [5,6,14,15]. The Government of Nigeria´s response to the coronavirus disease- 19 (COVID-19) pandemic has also benefited from the polio infrastructure and assets, similarly to other countries with polio-funded staff presence. The polio staff used their skills and experience to lead rapid response teams and utilized polio EOCs for COVID-19 while providing strategic leadership in the COVID-19 response. These examples indicate the successful transfer of skills and assets when the need arises. PEI has been associated with increased government spending on strengthening routine immunization systems and related improvements, especially in micro-planning, service delivery, supportive supervision program management and capacity building [16-18]. Similarly, the integrated disease surveillance system, data management and analysis capacity, and laboratory systems in Nigeria have also benefited from the polio-funded infrastructure, as reported here and for other countries in the WHO African Region [19-21].

With additional training and human resources at the EOCs, the functions can effectively be expanded for the long term to support other emergency responses, with expanded and contracted staffing as needed, VPD surveillance, and routine immunization strengthening. The expansion of roles of the Polio EOCs is being considered as part of the National Polio transition plan in Nigeria [7]. Our assessment is limited to three aspects. First, there were many other complimentary efforts made to systematically address the poor performance of the polio program in 2012 and later years, in addition to the establishment of the EOCs. We did not assess the contribution of other initiatives toward the interruption of WPV transmission. Secondly, due to the intensity of polio eradication activities and the occasional need for overlap of non-polio activities and campaigns, polio EOC staff were sometimes stretched with their primary responsibilities hindered the extent to which they could provide direct support to other public health programs, even though PEI field staff contributed substantially. NPHCDA eventually worked towards harmonizing a calendar of activities to ensure the overlap was minimized and that the use of available assets was maximized. In addition, polio EOC staff were able to provide mentoring and capacity-building based on experiences in the polio program. Thirdly, only 18 respondents were included in the assessment and participants were mostly internal to the polio program, which could have biased the views as against external respondents.

## Conclusion

The IMS under the EOC was felt by key informants to have strengthened coordination of government and partners towards achieving polio eradication in Nigeria, with benefits to other emergencies and the broader immunization system, including improving funding efficiencies. The EOC concept applies to managing effective support for various public health emergencies, such as disease epidemics, natural disasters, and humanitarian crises. The Government of Nigeria should maximize the infrastructure built at the polio EOCs, and their demonstrated capacity and experiences in managing emergencies and RI strengthening for long-term benefits to primary health care and health security.

**Disclaimer:** the findings and conclusions in this report are those of the authors and do not necessarily represent the official position of the U.S. Centers for Disease Control and Prevention.
